# Case report: Upper neck pouch sign in the antenatal diagnosis of esophageal atresia

**DOI:** 10.4103/0971-3026.54875

**Published:** 2009-08

**Authors:** Mukesh Kumar Garg

**Affiliations:** Department of Radiology, Geetanjali Medical College and Hospital, Udaipur, Rajasthan, India

**Keywords:** Esophageal atresia, upper neck pouch sign

## Abstract

Prenatal diagnosis of esophageal atresia remains a challenge for the imaging consultant. On antenatal USG, the finding of an absent or small stomach in the setting of polyhydramnios used to be considered suspicious of esophageal atresia. However, these findings have a low positive predictive value. The upper neck pouch sign is another sign that helps in the antenatal diagnosis of esophageal atresia. In this paper, I report a case of esophageal atresia that was diagnosed on USG at 27 weeks of gestation; the diagnosis was confirmed postnatally.

## Introduction

On antenatal USG, esophageal atresia is usually suspected when there is polyhydramnios and an absent or small stomach.[1] This, however, is not definitive; the positive predictive value of these findings is only 56%.[2] These findings may also be associated with other anomalies.[3] The presence of a blind-ending anechoic pouch in the fetal neck or mediastinum, the upper neck pouch sign, which is best visualized during fetal swallowing is an additional sign for the antenatal diagnosis of esophageal atresia.[4–6]

I report a case of esophageal atresia that was diagnosed antenatally at 27 weeks by demonstration of the upper neck pouch sign; the diagnosis was confirmed postnatally.

## Case Report

A 20-year-old multiparous (gravida 2) woman at 27 weeks' gestation was referred for a fetal well-being examination. There was no history of consanguinity and the family history was unremarkable. Her previous pregnancy had been uneventful.

**Figure 1:**
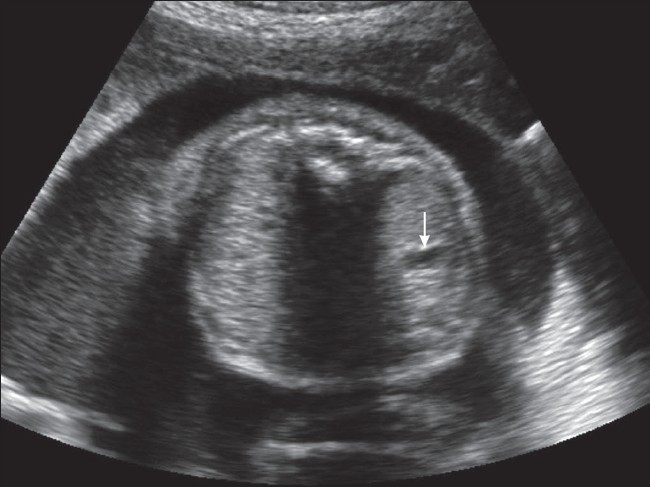
USG of the fetus in the transverse plane at the level of abdomen shows the small stomach (white arrow)

**Figure 2:**
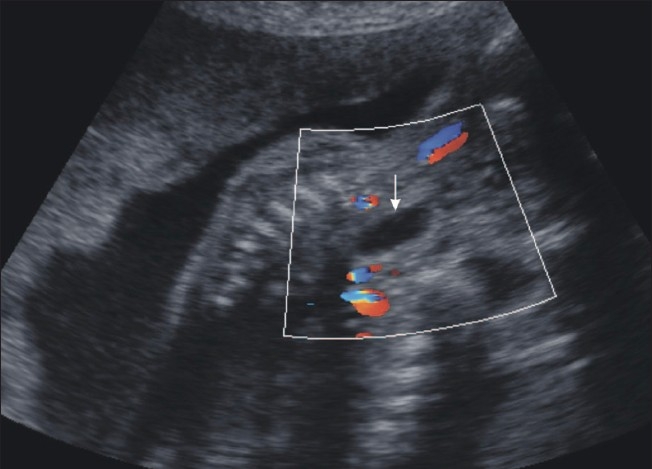
USG of the fetus in the sagittal plane at the level of neck shows an anechoic pouch (white arrow)

**Figure 3:**
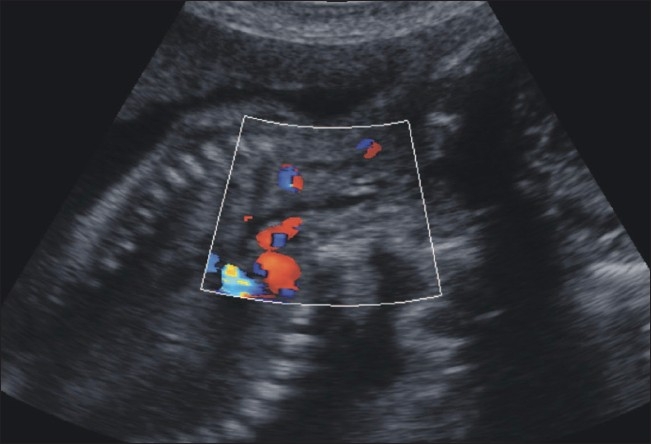
USG of the fetus in the sagittal plane at the level of neck shows no pouch (in the emptying stage)

Ultrasonography examination revealed polyhydramnios (amniotic fluid index: 25 cm) and a very small stomach [Figure 1]. In view of these findings, the fetal neck and chest were examined in detail. An anechoic, dilated, blind-ending proximal esophageal pouch was seen in the neck. It could be seen to be filling and emptying repeatedly on real-time examination [Figures 2 and 3]. Based upon this finding, a diagnosis of esophageal atresia was made. In view of the very small stomach, the presence of a distal tracheoesophageal fistula was suspected.

Ten days later the patient went into premature labor and delivered a male baby vaginally. The baby, however, died immediately after birth.

**Figure 4:**
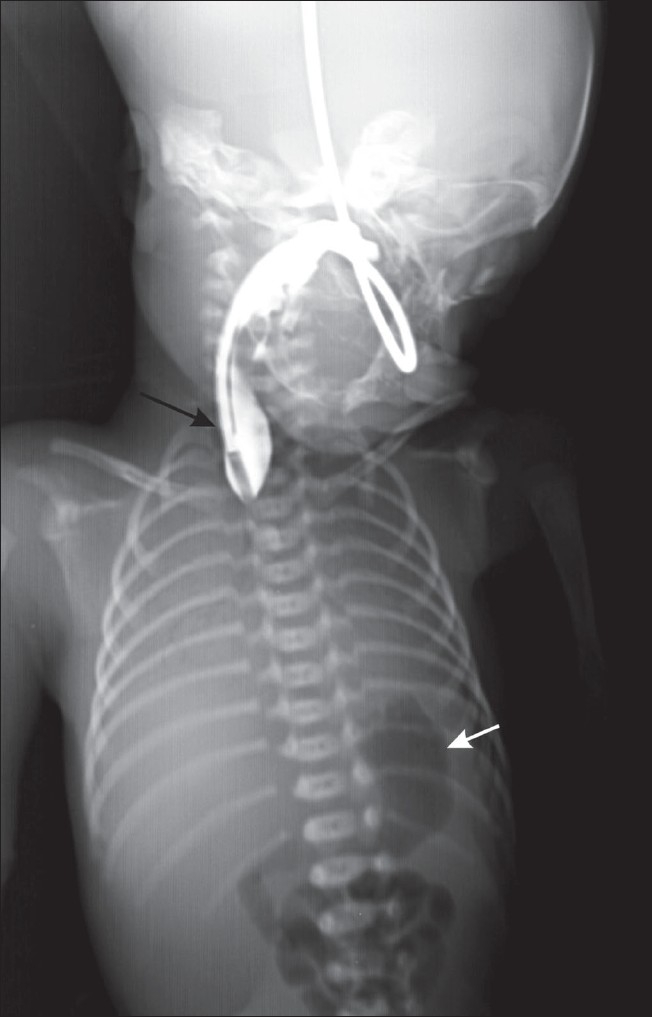
Chest radiograph of the baby with contrast shows the blindending upper end of the esophagus (black arrow) and the gas-filled stomach (white arrow)

**Figure 5:**
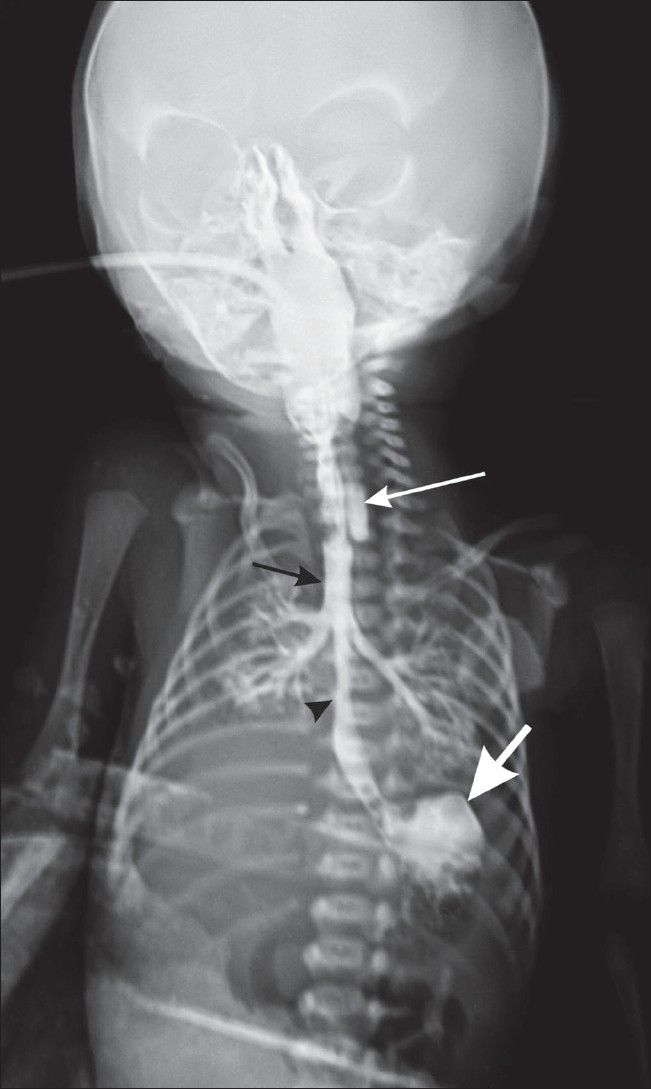
Chest radiograph of the baby with contrast shows the blindending upper end of the esophagus (long white arrow), contrast in the trachea (black arrow), the lower end of the esophagus communicating with the trachea (black arrow head) and contrast in the stomach (small, thick white arrow)

A postnatal radiograph of the chest with contrast in the esophagus revealed the blind-ending upper end of the esophagus [Figure 4]; the lower end of the esophagus was seen communicating with the trachea [Figure 5]. These findings confirmed the antenatal diagnosis of esophageal atresia with a distal tracheoesophageal fistula.

## Discussion

Esophageal atresia is a condition in which the proximal and distal portions of the esophagus do not communicate. The reported incidence is 1 in 3590 pregnancies.[7] Five types of esophageal atresia with tracheoesophageal fistula have been described;[8–10] in order of frequency, the common types are as follows: esophageal atresia with a distal tracheoesophageal fistula (87%),[10] isolated atresia (6%), ‘H-type’ tracheoesophageal fistula (4%), esophageal atresia with proximal and distal fistulas (2%) and esophageal atresia with a proximal fistula (1%). VACTERAL (vertebral, anorectal, cardiac, tracheal esophageal, renal and limbs) anomalies coexist in 50–70% of children with esophageal atresia.[12]

Antenatally, the diagnosis of esophageal atresia is suspected when USG reveals polyhydramnios along with an absent or small stomach. However, these findings are not conclusive. A moderately distended stomach may be visualized in a case of esophageal atresia with or without tracheoesophageal fistula as a consequence of retained or increased gastric secretions.[13] Polyhydramnios and an absent or small stomach may be associated with numerous other anomalies,[3] e.g., diaphragmatic hernia or deficient fetal swallowing due to mechanical obstruction, facial clefts or neuromuscular disease.

In our case, in addition to the presence of polyhydramnios and a very small stomach, an anechoic esophageal pouch was seen in the neck showing alternate filling and emptying on real-time examination. The pouch sign was described in 1995 as ‘a transient anechoic area in the midline of the fetal neck’[14] and was later named the ‘upper neck pouch sign’.[4]

To conclude, the presence of the upper neck pouch sign on USG is an additional sign that helps in the diagnosis of esophageal atresia; this sign is seen regardless of the presence or absence of a tracheoesophageal fistula.[4–6] When seen, the pouch is specific for this diagnosis, but is not appreciated in up to 57% of proved cases of esophageal atresia.[15] Therefore, in the presence of polyhydramnios, the radiologist should look for the pouch sign in the neck, irrespective of whether the stomach is present or absent.
